# A comprehensive cross-sectional survey to identify barriers and facilitators of cervical cancer screening in women with HIV in Guangxi, China

**DOI:** 10.1186/s13027-022-00426-2

**Published:** 2022-03-24

**Authors:** Ran Zhao, Shujia Liang, Deanna Teoh, Yunqing Fei, Xianwu Pang, Shalini Kulasingam

**Affiliations:** 1 Division of Epidemiology and Community Health, University of Minnesota School of Public Health, 300 West Bank Office Building, 1300 S 2nd St, Minneapolis, MN 55454 USA; 2 Institute of HIV Prevention and Control, Guangxi Zhuang Autonomous Region Center for Disease Control and Prevention, 18 Jinzhou Road, Nanning, 530028 Guangxi China; 3 Division of Gynecologic Oncology, University of Minnesota Department of Obstetrics, Gynecology and Women’s Health, 420 Delaware Street SE, MMC 395, Minneapolis, MN 55455 USA; 4 University of Minnesota Center for Global Health and Social Responsibility, C311 Mayo Building, 420 Delaware Street SE, Minneapolis, MN 55454 USA

**Keywords:** Cervical cancer screening, Women with HIV, “Knowledge attitudes and beliefs”

## Abstract

**Background:**

Co-infection with HIV is a strong risk factor for cervical cancer development. It is unknown whether women with HIV in Guangxi, China are utilizing currently available cervical cancer screening services, what barriers they face, and if they are aware of their increased risk of developing cervical cancer.

**Methods:**

Using a cross-sectional design, we administered a survey to women with HIV aged 21–65 years from August to October 2019 in Guangxi, China. A 100-item survey was designed in English and translated into Chinese. We assessed knowledge, attitudes, and beliefs about cervical cancer and cervical cancer screening, identified potential barriers and facilitators of cervical cancer screening programs for women with HIV, and assessed potential risk factors for cervical cancer.

**Results:**

A total of 101 participants completed the survey. The median age of participants was 38 years (IQR 34.5–44 years). Forty-seven percent of the women had been screened for cervical cancer at least once. The mean score was 5.6 out of 9 (95% CI 5.3–6.0) on the knowledge about cervical cancer and screening and 6.3 out of 10 (95% CI 5.9–6.6) for cervical cancer risk factors, respectively. Facilitators of participating in cervical cancer screening included trust and openness to healthcare workers having conversations about female health concerns. Barriers identified in our study included knowledge gaps in cervical cancer risk awareness and cervical cancer screening awareness, including the lack of knowledge of available cervical cancer screening services. Women with HIV in Guangxi are under-screened for cervical cancer.

**Conclusion:**

When designing tailored cervical cancer screening programs for women with HIV in Guangxi, educational programs to address existing knowledge gaps will be needed to increase screening uptake in this high-risk population.

## Background

Cancer of the cervix is the third most common cancer in females and the second leading cause of cancer death in women aged 15–44 years in China [1]. The cervical cancer incidence rate has been increasing over the last 20 years and is recognized as a critical public health problem in China [2]. Infection with human papillomavirus (HPV) is a necessary cause of cervical cancer [3]. Among all HPV types, HPV16, 18, 31, 33, 45, 52, and 58 are responsible for more than 90% of cervical cancers, with over 80% of all cases caused by HPV16 or 18 [4]. These HPV types can be effectively prevented by the HPV vaccines that are currently licensed in the world [5, 6]. Particularly, in China, recommendations of HPV vaccination target girls and women aged 9–45 years old [7].

HPV infections are common in the anogenital tract of women. Although the majority of women clear their HPV infections, certain risk factors, including tobacco smoking, high parity, and co-infection with human immunodeficiency virus (HIV), can increase the likelihood of progression from cervical HPV infection to cancer [8–12]. Co-infection with HIV is a strong risk factor for cervical cancer development. Due to a weakened immune system, women infected with HIV have a 4.1 times higher risk of developing cervical cancer [13]. Therefore, the World Health Organization (WHO) recommends cervical cancer screening for sexually active girls and women as soon as they have tested positive for HIV [14].

Routine cervical cancer screening followed by treatment is an effective strategy to reduce cervical cancer incidence. In 2009, the Chinese government launched the National Cervical Cancer Screening Program in Rural Areas (NCCSPRA) to address regional disparities in cervical cancer burden and serve as an initial step towards nationwide provision of population-based cervical cancer screening [15]. Current guidelines in China recommend screening for women aged 21–65 years with cytology every three years, an HPV DNA test every five years, or visual inspection with acetic acid and/or Lugol’s iodine (VIA/VILI) as an alternative to cytology in low-resource settings [7, 16]. Currently, eligible women can access screening at township-level hospitals [15]. However, the estimated uptake of cervical cancer screening among all eligible women is only 20% [15]. This low level of screening is worrisome for women with HIV despite their excess risk.

However, unlike the guidelines from other countries [17, 18], the current screening guidelines in China do not provide separate screening recommendations for women with HIV (e.g., shorter screening intervals, use of the more sensitive HPV test, or extended age to end screening) [15]. People with HIV usually receive HIV treatment at specialized clinics where cervical cancer screening is not among the routine services offered, while the health facilities offering cervical cancer screening don’t have access to the patients’ HIV infection status. It is unknown whether women with HIV in China are utilizing currently available cervical cancer screening services, what barriers they face, if they are aware of their increased risk of developing cervical cancer, or if they are knowledgeable about cervical cancer risk factors, HPV vaccination, and cervical cancer screening. In addition, information about established risk factors, including current CD4 cell count and exposure to smoking or secondhand smoke (SHS), can help inform the design of tailored screening programs to reduce the incidence of cervical cancer in this high-risk group of women.

Guangxi is a region in China with a high prevalence of HIV infection. Its HIV prevalence is three times the national average (1.5% vs. 0.45%) [19]. From 2010 to 2017, over 85,000 newly diagnosed HIV infections in women were reported to the Guangxi Center for Disease Control and Prevention (CDC) [20]. According to the 2015–2016 surveillance data, the most common route of HIV infection among women changed from injection drug use to sexual transmission [21]. Both HPV and HIV are sexually transmitted infections and can be co-transmitted, putting women with HIV at a high risk of incident and persistent HPV infections and subsequent cervical cancer [13]. The current study aims to assess the knowledge, attitudes, and beliefs about cervical cancer, as well as the barriers and facilitators of cervical cancer screening in women with HIV in Guangxi, China.

## Methods

### Study design

This study was approved by the University of Minnesota and the Guangxi CDC Institutional Review Boards. Using a cross-sectional design, we recruited women with HIV from the Guangxi CDC HIV clinic from August to October 2019. This clinic provides specialized HIV treatment to infected women in Nanning, the capital of Guangxi province. Based on the current screening recommendations, women were eligible for the study if they were 21–65 years old. The HIV treatment center staff made initial contact with potential participants during their routine treatment visits and introduced the project to them. Patients were informed about the objectives of the study and had an opportunity to ask study-related questions. All patients who were willing to participate provided written informed consent. Women who consented to participate and completed the interviews received compensation of 50 Chinese Yuan (CNY) (approximately 7.7 USD) for their time.

### Data collection

The survey was developed in English and translated to Chinese by experts with specialized knowledge of medical terminology. The survey had eight sections, including I. demographic information, II. sources of information about cervical cancer, III. knowledge about cervical cancer, screening and HPV vaccination, IV. knowledge about risk factors for cervical cancer, V. beliefs about cervical cancer and screening, VI. barriers and facilitators of cervical cancer screening, VII. sexual history and pregnancy history, and VIII. exposure to smoking and SHS. The majority of the survey items were close-ended. Trained interviewers from the Guangxi CDC administered the survey in a designated room to ensure confidentiality. Clinical information on each participant, including the confirmation date for the HIV diagnosis, CD4 cell count, and viral load from the most recent examination, were retrieved from the Guangxi CDC HIV epidemic database. Abstracted data were independently entered into EpiData Entry version 3.1 (The EpiData Association. Odense Denmark, 2002.) by two data entry staff and checked and corrected for any discrepancies by a third study staff member.

### Data analysis

We evaluated the responses of the two knowledge-based sections (i.e., III. knowledge about cervical cancer, screening, and vaccination and IV. knowledge about cervical cancer risk factors) by comparing the participant answers to an answer key. Each correct response contributed one point. For items with multiple correct responses, a full point was given if the participant correctly identified at least one right answer. The maximum total points a participant could get was nine for section III and ten for section IV. We summarized continuous responses (e.g. age, CD4 cell count, time since HIV diagnosis, monthly family income, the number of household residents, pregnancies, live-births, lifetime and current sex partners, time to get to the health facility, and the total points of sections III and IV) using the median and the interquartile range (IQR). The rest of the survey responses were categorical and were summarized as the total number (n) and the percentage (%) in each category. Statistical analyses were conducted using Stata version 15.1 (StataCorp. 2017. *Stata Statistical Software: Release 15*. College Station, TX: StataCorp LLC.). Missing responses were omitted in the final analysis.

## Results

### Demographics

Ninety-one percent of women with HIV we approached agreed to participate in the study and provided written informed consent. We enrolled 101 women with HIV who were receiving treatment at the CDC HIV clinic. Demographic information about the population is summarized in Table 1. The median age among participants was 38 years (IQR: 34.5–44 years old). The median CD4 cell count was 654 cells/µl (IQR: 493–854 cells/µl). The median monthly family income was 2500 CNY (equivalent to 375 US Dollar) (IQR: 1900–4000 CNY). Eighty percent of the women completed middle school (equivalent to nine years of education), and 87% were employed. Within the past 30 days, 86% of women were exposed to SHS either at home or at work.

**Table 1:** Demographics of survey participants

Variable	Mean	Median	IQR	n	%
Age (year)	39.5	38	34.5–44		
CD4 cell count (cells/µl)	705.0	654	493–854		
Time since HIV diagnosis (year)	8.7	9	6–12		
Monthly family income (CNY)		2500	1900–4000		
Number of people living in the household	3.1	3	2–4		
Education					
< Middle school				20	19.8
Middle school				50	49.5
High school				23	22.8
Some college				8	7.9
Graduate school or higher				0	0.0
Partner status					
Single				4	4.0
Married				78	77.2
Widowed				7	6.9
Divorced				12	11.9
Have been screened for cervical cancer				47	46.5
Have received HPV vaccine				4	4.0
Occupation					
Agriculture				15	14.9
Business				2	2.0
Housewife				13	12.9
Government worker				0	0.0
Service industry				36	35.6
Other				35	34.7
SHS exposure at home					
Daily				62	61.4
Weekly				6	5.9
Monthly				1	1.0
Less than monthly				1	1.0
SHS exposure at work last 30 days				72	71.3

### Sexual and pregnancy history

Eighty-six percent of women had used some method to delay or avoid getting pregnant. These methods included condoms (77%), contraceptive pills (31%), intrauterine devices (IUD) (20%), and female sterilization (11%). The median number of pregnancies was 3.0 (IQR: 2–4), with a median number of live births of 1(IQR: 1–2). The median reported number of lifetime sexual partners was 2 (IQR: 1–3). The median reported age of sexual debut was 21 years (IQR: 20–23 years old).

### Knowledge of cervical cancer

Knowledge of cervical cancer is presented in Table 2. A majority (94%) of women had heard of cervical cancer, and 28% knew someone who had been diagnosed with cervical cancer. The most common sources of cervical cancer-related information were television (67%), the internet (66%), friends or family members (55%), and health facilities (54%). The mean score of knowledge about cervical cancer and cervical cancer screening was 5.6 (range: 1–9; 95% CI 5.3–6.0) out of nine. Seventy percent of women believed that cervical cancer could be prevented. Seventy-one percent of women correctly identified screening as a potential preventive method. However, 9% of women were not aware of any preventive strategies, and two women believed that nothing could be done to prevent cervical cancer.

**Table 2:** Knowledge of cervical cancer

Variable	n	%
Heard of cancer	98	97
Heard of cervix	93	92.1
Heard of cervical cancer	95	94.1
Know someone diagnosed with cervical cancer	28	27.7
Source of information about cervical cancer		
Radio	9	8.9
TV	68	67.3
Internet	67	66.3
Poster	19	18.8
Health facility	55	54.5
Friends/family members	56	55.5
Newspaper/magazines	41	40.6
School	0	0
Scoring items	n	**%**
Do you think cervical cancer can be prevented?		
Yes	71	70.3
No	8	7.9
Don't know	22	21.8
What cervical cancer preventive measures do you know about?		
Screening	72	71.3
Not smoking	40	39.6
Vaccination	72	71.3
Other	0	0
Don't know	9	8.9
None	2	2
What are some of the symptoms of cervical cancer?		
Irregular bleeding	64	63.4
Bleeding after sex	55	54.5
Bleeding after menopause	57	56.4
Weight loss	33	32.7
Foul smelling vaginal discharge	74	73.3
Abdominal/pelvic/back pain	42	41.6
Other	5	5
No symptom	8	7.9

### Knowledge of cervical cancer prevention

Knowledge of cervical cancer prevention is presented in Table 3. Seventy-seven percent of women identified either Pap test, HPV test, pelvic test, or direct visual inspection as methods for detecting cervical cancer and its precursor lesion. However, 23% of women were unaware of any test to detect cervical cancer. Fifty-seven percent of women believed that cervical cancer is curable if it is limited to the cervix. For cervical cancer screening-related questions, none of the participants knew the recommended screening start age of 21 years. Seventy-five percent of women thought screening frequency should be once a year. For vaccine-related questions, 74% thought women could be vaccinated against the virus that causes cervical cancer. However, the participants preferred vaccinating adults instead of vaccinating adolescents (9% for < 18 years old vs. 66% for 18 years or above).

**Table 3:** Knowledge of cervical cancer prevention

Variable	n	%
Which tests can be carried out to detect cervical cancer?		
Pap test	74	73.3
Direct visual inspection	17	16.8
HPV test	66	65.4
Pelvic exam	21	20.8
Don’t know	23	22.8
Do you think that cervical cancer is curable if it is found to be limited to the cervix?		
Yes	58	57.4
No	26	25.7
Don't know	17	16.8
At what age should a woman start screening for cervical cancer?		
< 21 years old	18	17.8
At 21 years old	0	0
> 21	76	76.3
Don't know	7	6.9
How often should a woman be screened for cervical cancer?		
At least once a year	86	85.2
Every two years	3	3
Every three years	4	4
Don't know	8	7.9
Can someone be vaccinated against the virus that causes cervical cancer?		
Yes	75	74.3
No	14	13.9
Don't know	12	11.9
At what age should a person receive a vaccination to prevent cervical cancer?		
< 9 years old	0	0
9–45 years old	75	75.3
> 45 years old	0	0
Don't know	25	24.8

### Knowledge of cervical cancer risk factors

**Fig. 1:**
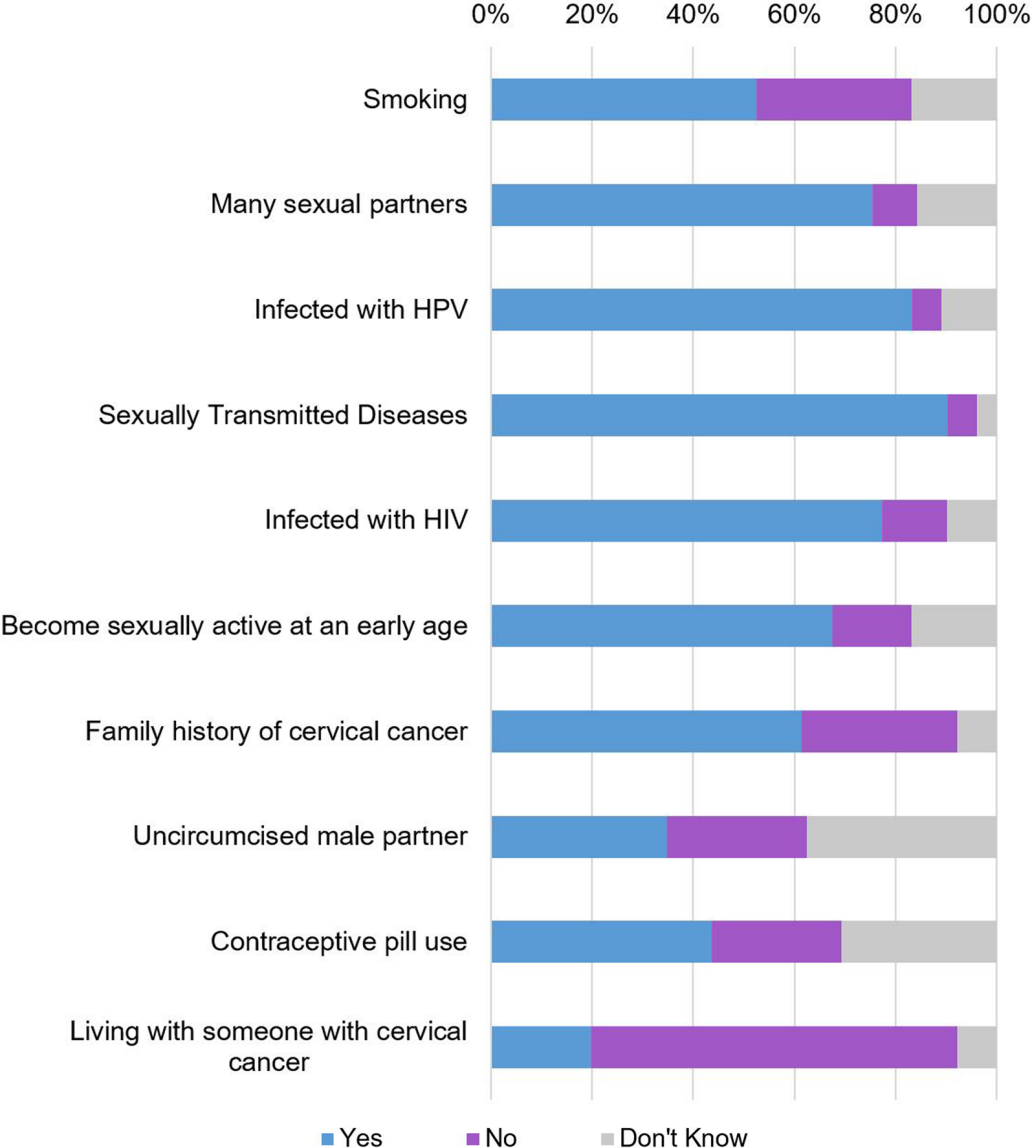
Knowledge of cervical cancer risk factors

The mean score for knowledge of cervical cancer risk factors was 6.3 (range: 2–10; 95% CI 5.9–6.6). The responses for knowledge of each survey item are presented in Fig. 1. The most well-known risk factors for cervical cancer were sexually transmitted infections (90%), followed by HPV infection (83%), HIV-coinfection (77%), having many sexual partners (75%), early sexual debut (67%), and smoking (52%). Sixty-one percent and 20% of women falsely identified family history and living with someone with cervical cancer as risk factors, respectively.

### Attitudes, beliefs, and acceptance of screening and vaccination

**Fig. 2:**
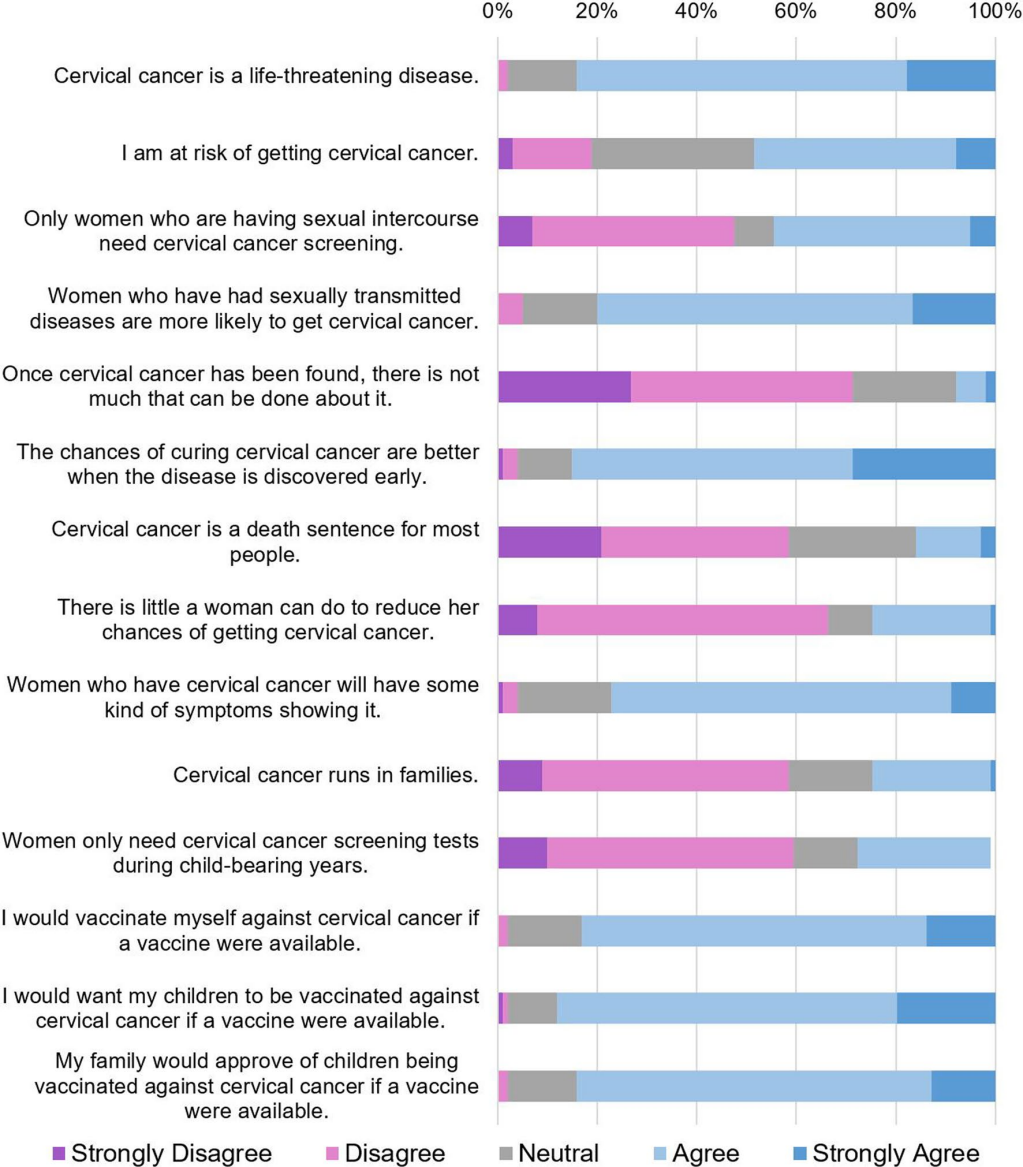
Beliefs towards cervical cancer, screening, and vaccination

Forty-seven percent of women had been screened for cervical cancer at least once in their lifetime, and 4% said they had received the HPV vaccine. The responses for beliefs about cervical cancer, cervical cancer screening, and vaccination are summarized in Fig. 2. Eighty-four percent of women agreed that cervical cancer is a life-threatening disease, but only 49% thought that they were at risk for cervical cancer. Forty-five percent of women believed that only women who were currently having sexual intercourse needed cervical cancer screening, and 27% thought that cervical cancer screening was only necessary during child-bearing years. Seventy-one percent of women believed that cervical cancer was a manageable disease, and 84% felt that the chances of curing cervical cancer were better when the disease was discovered early. Eighty-four percent of women favored personally receiving the vaccine against cervical cancer, and 87% would want their children to receive the vaccine against cervical cancer.

### Potential barriers and facilitators

We summarize the results of potential barriers and facilitators of cervical cancer screening in Table 4. When experiencing health problems, 91% of women reported going to local health facilities. The median time to get to the health facility was 15 min (IQR: 10–30 min). Twenty-five percent of women reported that their usual healthcare facilities did not offer cervical cancer screening. Seventeen percent of women did not know if their healthcare facility provided screening services, and among them, 93% expressed that they would be willing to be screened if they were told that their local healthcare facility provided screening services. Eighty percent of women were willing to self-test at home if a self-collected test were available. Almost all women (99 out of 101) felt comfortable talking to healthcare workers about women’s health issues. All women had mobile phones capable of receiving text messages, but only 13% were willing to receive text messages about cervical cancer-related information.

**Table 4:** Barriers to Cervical Cancer Screening

Variable	n	%
To where do you go when you have a health problem?		
Local healthcare facility	92	91.1
HIV clinic	2	2.0
Other	7	6.9
How do you usually get to the local healthcare facility/HIV clinic from home?		
Own car	17	16.8
Taxi	3	3.0
Car rideshare	5	5.0
Electric moped	42	41.6
Walking	12	11.9
Public transportation	18	17.8
Other	4	4.0
How long do you usually wait to be seen at the clinic?		
< 10 min	13	12.9
10–30 min	40	39.6
30–60 min	35	34.7
> 60 min	13	12.9
Is screening for cervical cancer offered at the local healthcare facility/HIV clinic?		
Yes	59	58.4
No	25	24.8
Don't know	17	16.8
If screening for cervical cancer were offered at the local healthcare facility/HIV clinic, would you go to the clinic to be screened?		
Yes	39	92.9
No	2	4.8
Don't know	1	2.4
If you could have a test that is available to you at home, would you like to take the test?		
Yes	81	80.2
No	20	19.8
Do you feel comfortable talking to the healthcare workers at the clinic about women's health issues?		
Yes	99	98.0
No	2	2.0
If you have children, who takes care of them when you go to the clinic?		
Family members	39	38.6
Childcare	11	10.9
Other	25	24.8
Nobody	26	25.7
Do you need to get permission from a family member to be able to get healthcare?		
Yes	2	2.0
No	99	98.0

## Discussion

In our study population, 47% of the participants had been screened for cervical cancer at least once in their lifetime. This coverage is higher than the estimated 20% screening coverage among Chinese women [15]. However, the proportion of women with HIV who are screened at the recommended interval of every three years is likely lower than 47%. Second, the higher screening uptake could be associated with the potential facilitators we identified among women with HIV in Nanning, China.

First, a majority of the survey respondents did not need family permission to obtain health care when needed. Second, women with HIV reported trusting relationships with healthcare workers. The majority of the patients expressed trust and a willingness to talk about women’s health problems with healthcare workers and were willing to be screened for cervical cancer if they were told that the services were available. Third, the median time for the participants to get to the health facility was only 15 min, which suggests that proximity to screening services could help facilitate participation. In addition, most women did not need to worry about taking care of their children when they needed health care. As such, women with HIV in our study may be well placed to participate in cervical cancer screening when screening programs are available. However, given the excess risk for developing cervical cancer in women with HIV, further improving screening coverage is urgently needed.

Previous studies have identified both individual- and geographic-level factors contributing to the low screening coverage among the general population. In addition to fear of screening outcomes, cultural barriers for intimate examinations, income, education, and geographic regions, gaps in knowledge and health awareness and limited information on cervical cancer are some critical barriers to participating in cervical cancer screening among Chinese women [22–24]. Our cross-sectional study has similar findings for women with HIV in Guangxi. We identified several knowledge gaps and potential areas for improvement in this population.

First, there was a knowledge gap in cervical cancer risk awareness. Half of the women with HIV did not agree with the statement that they themselves were at risk of cervical cancer, and many believed that screening was only needed during child-bearing years. This contrasts with recommendations that the target population for cervical cancer screening is between the ages of 21 and 65 years, and that the risk of cervical cancer peaks around age 45 [25]. Although women with HIV were knowledgeable about HIV increasing the risk of cervical cancer, they were not able to link that knowledge to themselves of having high-risk for cervical cancer, underscoring the urgent need for education programs in this population.

Second, there was a knowledge gap in cervical cancer prevention strategies. Although the majority of women identified the Pap test as a screening test for cervical cancer, none of them knew the recommended screening start age and the screening interval. Moreover, only 4% of the participants felt that the vaccination should be given to girls aged 9–14 years old, which is the primary target age group of the HPV vaccine.

Third, there was a gap in knowledge about available cervical cancer screening services. In China, women aged 35–64 years who reside in rural areas are eligible for free cervical cancer screening. Women who are not eligible for the free screening program can still receive screening at the township-level hospitals [15]. Among women with HIV who did not know that local health facilities provide cervical cancer screening, almost all of them would be willing to be screened if they knew screening was offered at the local health facilities. These results indicate that women with HIV were not familiar with the current cervical cancer screening services available to them.

The knowledge gaps we identified could be associated with the lower utilization of available screening services. To increase screening coverage in this high-risk population, an education program could be implemented. Such an education program could address the connection between being infected with HIV and the increased risk of cervical cancer, which will ideally increase health awareness among women with HIV. The program should also aim to increase awareness regarding available cervical cancer screening services offered at the local health facilities. One potential strategy to achieve these aims is to hand out pamphlets with information about local health facilities that provide cervical cancer screening services, the target age group, and the recommended screening interval. Such efforts could potentially increase the utilization of available screening services.

To further increase screening coverage, tailored screening programs for women with HIV should be developed and implemented. Such programs should consider providing screen services at a more frequent interval, using tests with increased sensitivity, and/or extending the screening end age in order to account for the extended period over which women with HIV are at risk for cervical cancer [17, 18]. Tailored screening programs would ideally be located in the HIV treatment centers and screening performed at the time of HIV surveillance visits, where women are seen every three months, increasing the likelihood of more women being screened with the recommended screening schedules. Patients who are diagnosed with pre-cancerous and malignant lesions can be referred to healthcare facilities that provide treatment and surveillance recommendations.

Finally, our data suggest that the majority of women with HIV in Guangxi have achieved viral suppression. However, prevalent exposure to SHS is a potential risk factor for cervical cancer among women with HIV in Guangxi. Over 85% of women were exposed to SHS in the past 30 days. To our knowledge, no studies have evaluated the effect of exposure to SHS on cervical cancer development among women with HIV. Given the high SHS exposure in Chinese women with HIV, future studies are needed to determine whether SHS exposure is a risk factor for cervical cancer development, so that this additional risk can be addressed through smoking prevention programs when designing a tailored screening program.

Our study has several limitations. We enrolled 101 participants from one HIV treatment clinic in Nanning, Guangxi. This clinic provides specialized HIV treatment to 3.4% of women with HIV in Guangxi. Nanning is an urbanized city in Guangxi. Given the regional disparities in cervical cancer disease burden [15], the importance of addressing the knowledge gaps in the entire region of Guangxi is probably more crucial. Future studies that enroll participants from other clinics may provide a more comprehensive picture of cervical cancer screening in this region. In addition, the majority of questions in our survey were adapted from published studies [26]. As such, the validity and reliability of the survey in a Chinese population have not been evaluated. Finally, we focused on obtaining descriptive results from our study population as a first step towards being able to understand screening in this high-risk population. To ensure that the facilitators and barriers we identified in the studies are truly associated with participating in cervical cancer screening, further analyses will need to account for potential biases such as confounding effects.

Our study also has several strengths. Our study focuses on women with HIV. Given the low cervical cancer screening coverage among Chinese women, efforts to promote cervical cancer screening are required. With limited resources, such efforts could be most effective if targeted at high-risk populations, such as women with HIV. Our survey assessed a variety of topics related to cervical cancer in women with HIV. This information is critical for designing tailored screening programs for women with HIV in China.

## Conclusion

Women with HIV in Guangxi, China, who are at especially high risk of developing cervical cancer, are under-screened for cervical cancer. Our study identified important knowledge gaps in health awareness, cervical cancer, and access to cervical cancer screening. When designing tailored cervical cancer screening programs for women with HIV in Guangxi, the knowledge gaps identified in our study will need to be addressed to increase screening in this high-risk population.

## Data Availability

The datasets used for the current study are available from the corresponding author upon request, with justification as needed.

## Abbreviations

CDC: Center for Disease Control and Prevention
CNY: Chinese Yuan
HIV: Human immunodeficiency virus
HPV: Human papillomavirus
IUD: Intrauterine device
NCCSPRA: The National Cervical Cancer Screening Program in Rural Areas
SHS: Secondhand smoke
VIA/VILI: Visual inspection with acetic acid and/or Lugol’s iodine
WHO: The World Health Organization
