# Correction: A comprehensive cross-sectional survey to identify barriers and facilitators of cervical cancer screening in women with HIV in Guangxi, China

**DOI:** 10.1186/s13027-022-00437-z

**Published:** 2022-05-11

**Authors:** Ran Zhao, Shujia Liang, Deanna Teoh, Yunqing Fei, Xianwu Pang, Shalini Kulasingam

**Affiliations:** 1 Division of Epidemiology and Community Health, University of Minnesota School of Public Health, 300 West Bank Office Building, 1300 S 2nd St, Minneapolis, MN 55454 USA; 2 Institute of HIV Prevention and Control, Guangxi Zhuang Autonomous Region Center for Disease Control and Prevention, 18 Jinzhou Road, Nanning, 530028 Guangxi China; 3 Division of Gynecologic Oncology, University of Minnesota Department of Obstetrics, Gynecology and Women’s Health, 420 Delaware Street SE, MMC 395, Minneapolis, MN 55455 USA; 4 University of Minnesota Center for Global Health and Social Responsibility, C311 Mayo Building, 420 Delaware Street SE, Minneapolis, MN 55454 USA

## Correction to: Infectious Agents and Cancer (2022) 17:12. 10.1186/s13027-022-00426-2

Following publication of the original article [1], the authors identified a missed in-text citation to the original reference 26 (Mukama T, Ndejjo R, Musabyimana A, Halage AA, Musoke D. Women’s knowledge and attitudes towards cervical cancer prevention: a cross sectional study in Eastern Uganda. BMC Womens Health. 2017;17(1):1–8). A citation for this reference should be added in the ‘Methods’ section, in the first sentence of the ‘Data collection’ sub-section.

The original article [1] has been corrected: the missed citation in the ‘Data collection’ subsection has been added, reference 26 has been renumbered to reference 22 and the subsequent references and their citations have been renumbered as well.

## References

[1] Zhao (2022). A comprehensive cross-sectional survey to identify barriers and facilitators of cervical cancer screening in women with HIV in Guangxi, China. Infectious Agents and Cancer.

